# Fake science: The impact of pseudo-psychological demonstrations on people’s beliefs in psychological principles

**DOI:** 10.1371/journal.pone.0207629

**Published:** 2018-11-27

**Authors:** Yuxuan Lan, Christine Mohr, Xiaomeng Hu, Gustav Kuhn

**Affiliations:** 1 Department of Psychology, School of Social Sciences, Tsinghua University, Beijing, China; 2 Department of Psychology, Lausanne University, Lausanne, Switzerland; 3 Department of Psychology, Goldsmiths University of London, London, United Kingdom; Middlesex University, UNITED KINGDOM

## Abstract

Magicians use deception to create effects that allow us to experience the impossible. More recently, magicians have started to contextualize these tricks in psychological demonstrations. We investigated whether witnessing a magic demonstration alters people’s beliefs in these pseudo-psychological principles. In the classroom, a magician claimed to use psychological skills to read a volunteer’s thoughts. After this demonstration, participants reported higher beliefs that an individual can 1) read a person’s mind by evaluating micro expressions, psychological profiles and muscle activities, and 2) effectively prime a person’s behaviour through subtle suggestions. Whether he was presented as a magician or psychologist did not influence people’s beliefs about how the demonstration was achieved, nor did it influence their beliefs in pseudo-psychological principles. Our results demonstrate that pseudo-psychological demonstrations can have a significant impact on perpetuating false beliefs in scientific principles and raise important questions about the wider impact of scientific misinformation.

## Introduction

Magicians use deception and illusions to create effects that allow us to experience the impossible [1] by creating a cognitive conflict between the things we witness and the things we believe possible [2–4]. Magicians’ conjuring techniques can extend the boundaries of what we are willing to believe, and throughout history, magicians have used these techniques to push the boundaries of what we believe possible [5]. For example, ancient Egyptian priests used conjuring tricks to create the illusion of communicating with deities [6], Victorian spiritualists staged séances that fuelled beliefs in the spiritual underworld [7], and more recently, magicians have helped perpetuate beliefs in psychic powers [8]. Today, magicians have embraced a new story-telling “packaging”, also known as pseudo-explanations [9], by making reference to pseudo-psychological phenomena.

Derren Brown is one of the UK’s most successful magicians. He has developed a form of magic that blends trickery and psychology. He proclaims that his performances work, because he uses “Mind Control”. For example, in one of his performance pieces, Derren Brown claims to use unconscious primes to control an individual’s mind, which enables him to predict, with extremely high certainty, a “freely” chosen object by this individual. This explanation, however, is the story-telling “packaging”. Derren Brown uses conventional conjuring techniques to demonstrate scientifically implausible psychological phenomena or exaggerates the effects of established ones. In the past, magicians claimed to read your mind by contacting spirits; today they use the same tricks, but claim to use unconscious primes or to read your body language, and micro-expressions. Unlike most fraudulent psychics, these magicians do not deny the use of tricks; it is rather the observers themselves who often struggle to distinguish fact from fiction [10, 11] and accept ideas beyond false solutions [12]. In the end, the demonstrations are meant to entertain, yet these magicians may influence public understanding of psychology, and perpetuate pseudo-psychological beliefs.

Most magicians do not intentionally aim to misinform the public, and throughout history, magicians have helped uncover fraudulent spiritualists’ claims. Most magicians are honest about their deception. They typically tell the audience that what they are seeing are simply magic tricks. The label “entertainment magician” implies that what you are seeing is simply a trick and thus not real [3]. However, previous research shows that people often struggle to distinguish between “real magic” and trickery [13–15]. Knowing that they are witnessing a magic trick may not necessarily protect people from the misinformation that is part of the performance itself. Framing a magic performance as a psychological demonstration may therefore inadvertently help perpetuate false beliefs about psychology.

Empirical studies, indeed, show that the experience of a magic performance can impact our cognitive and affective functioning. For example, Subbotsky [16] showed children at and below 9 years of age a magic trick in which a magic spell caused a stamp to be burned and scratched. Prior to seeing the trick, most of the older children denied the existence of real magic, but after witnessing the trick, the majority endorsed this magical belief. The older children regained their sceptical view once they were told how the trick was done, but the younger children continued to believe in magic, even though they knew it was a trick. These results suggest that anomalous experiences can change children’s beliefs (see also [17]).

Adults’ explicit magical beliefs, on the other hand, seem more resilient to change. Mohr et al. [14] exposed participants to a magic trick that was framed as a demonstration in psychic powers, which did not change adult’s paranormal beliefs. In a series of recent follow-up experiments, Lesaffre et al. [15] staged psychic demonstrations, which many of the participants attributed to genuine spiritual forces. Yet, these demonstrations did not alter people’s general paranormal beliefs. However, adults may be prone to changing their beliefs in impossible phenomena when presented with scientifically plausible pseudo-explanations. Subbotsky [18] asked participants to watch an object magically transform. They were told that the transformation was either caused by a magic spell, or an electronic device. Although there was no logical explanation as to how the device could transform the object, participants were more willing to endorse an impossible, yet scientific plausible explanation than a magical explanation. We conclude that whilst adults may be resistant to changing their explicit paranormal beliefs, pseudo-scientific beliefs may be malleable.

We tested this latter possibility in the current experiment. More concretely, our study had two objectives. The first aim tested whether a magic demonstration using “psychological skills” changes people’s belief in the performer’s pseudo-psychological skills and the principles more generally. The second aim tested whether the performer alters people’s interpretation of the phenomena as a function of whether the performer was introduced as a magician or a psychologist. We targeted the first aim by having participants watch a demonstration in which the performer claimed to use psychological skills to read a volunteer’s thoughts. The volunteer was asked to secretly conceal a coin in his right or left hand. The performer claimed to use psychological skills to identify the coin’s location. Whilst it is possible to read aspects of a person’s mind by observing behaviour, these techniques are generally unreliable. Our performer, indeed, used a conjuring device to accurately deduce the coin’s physical location.

We targeted the second aim by informing half of the sample that the performer is a magician and the other half that he is a psychologist. When considering entertainment magicians, they often use disclaimers that inform the audience that what they are seeing is not real. Magic is based on lies and deception, and knowing that one is watching a magician rather than a psychologist should make the respective person more sceptical. Previous studies, indeed, showed that disclaiming a performance as a magic or psychic performance resulted in correspondingly higher magic or psychic interpretations of anomalous experiences [13, 14]. Such disclaimers seemed, however, inefficient when witnessing magic performances depicting a convincingly staged paranormal event [15]. Here, the authors found no differences in how participants explained the event being performed, regardless of whether they were informed that the demonstration was conducted by a true psychic or a magician who uses tricks and deception. What was observed instead, was that psychic explanations were as likely as conjuring explanations, and about 30% of the participants considered that both psychic and magic phenomena can explain the events, irrespective of what they had learned about the performer [15]. Thus, we could expect that the contextualization (performer is a magician versus psychologist) would have no impact on how participants explain the event.

## Method

### Participants

We recruited 90 undergraduate students who enrolled for a psychology degree program at Tsinghua University (37 males). The sample size was based on a previous study that used a similar design [14]. Our sample had a mean age of 19.6 years (SD = 1.2). All of the students attended a lecture on Psychology. The study was conducted in Mandarin. The study was approved by the Psychology department’s ethics board (Tsinghua University), and each participant provided written informed consent prior to the experiment.

### Material and procedure

Magic performance: The magic demonstration was performed by an amateur magician (first author) who is a member of Tsinghua University Magic Association. The magician asked to use the lecturer as a volunteer. She was asked to place a coin in either the left or right hand without the magician being able to see where she put the coin. When returning to the lecturer, the magician claimed to be using suggestion, psychology, micro expressions and muscle reading, to determine the correct location of the coin (see S1 Text for full description). On four out of four occasions, the magician correctly identified the coin’s location.

Belief in Psychological Principles Questions (BPPQ): We formulated 15 questions asking about people’s beliefs that a performer can use psychological principles to succeed in magic performances (see S2 Text). The questions targeted five principles: suggestion, personality-based prediction, microexpressions, muscle reading (i.e. ideomotor), and mind reading (see appendix). To target all principles, we formulated for each principle three BPPQ targeting i) belief in actual principle (BPPQ Belief), ii) principle was used in the current demonstration (BPPQ Used), and iii) principle can be applied in other situations too (BPPQ General). Participants rated each question using a 7-point Likert scale, ranging from 1 (strongly disagree) to 7 (strongly agree). The questions were presented in a random order. We calculated the mean BPPQ Belief, BPPQ Used, and BPPQ General scores (see supplementary material for actual questions).

Event interpretation questions: Participants were asked how they thought the demonstration had been achieved. Using a 7-point Likert scale ranging from 1 (strongly disagree) to 7 (strongly agree), they were asked whether the demonstration was accomplished through (1) paranormal, psychic or supernatural powers (psychic explanation), (2) ordinary magic trickery (trickery explanation), (3) religious miracles (religious explanation) or (4) psychological skills (psychological explanation) (see also [14]).

#### General procedure

**Fig 1:**
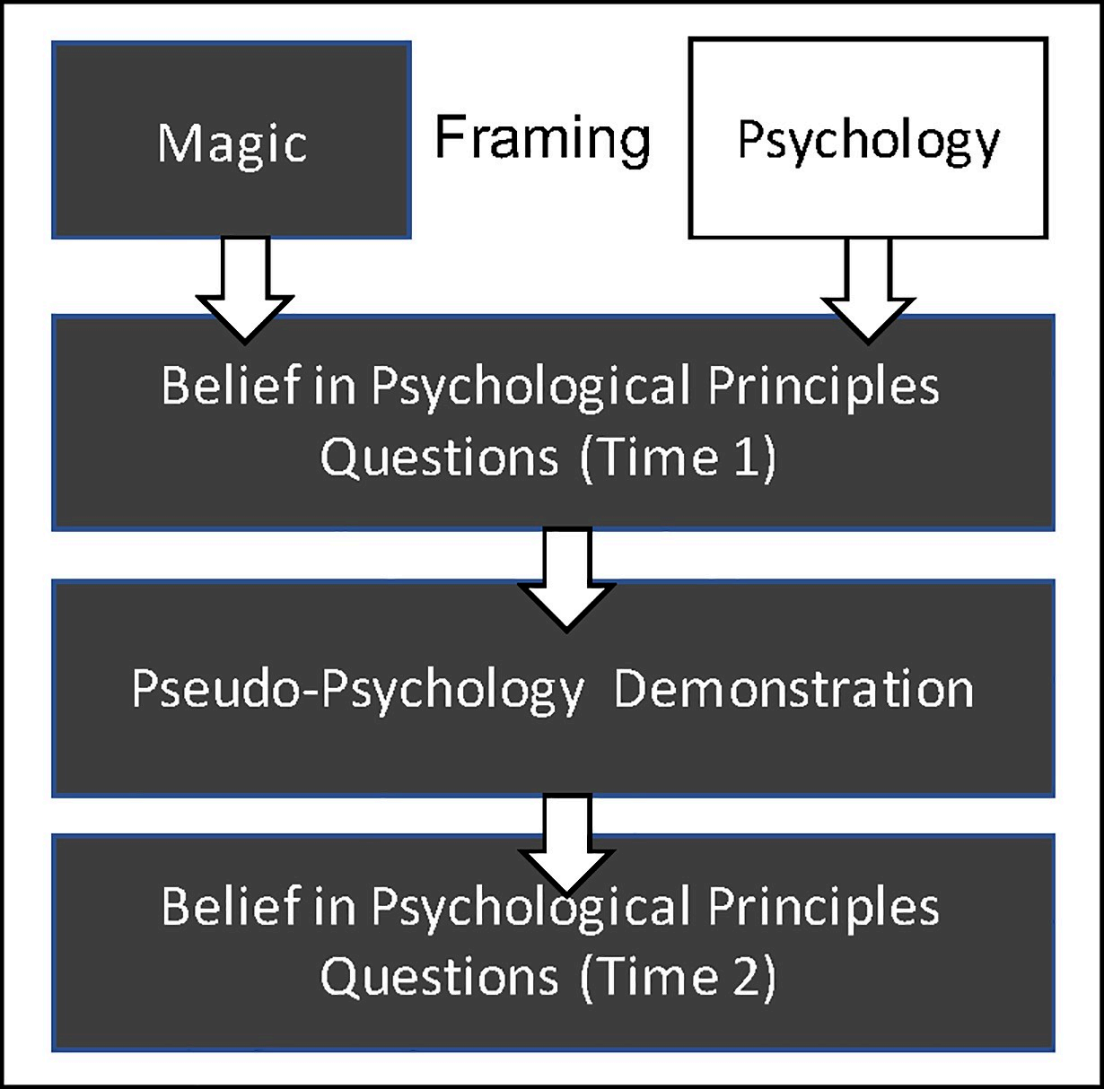
Flow diagram of the procedure.

The study was conducted in a large lecture theatre following a set sequence of events (see Fig 1). All of the testing occurred in one session. The female lecturer told her students that they were participating in an experiment on mental magic. Participants were then asked to use their smartphones to navigate to a website that provided additional information about the study. This website was also used to collect the behavioural data. The website randomly allocated participants to the psychology or magic framing condition group. Participants in the magic group were informed that the study was initiated by the Magic Association on four occasions, and that the demonstration was conducted by a member of a Magic Association. Participants in the psychology group were given the identical information apart from the fact that the word Magic Association was replaced by Psychology Association (see supplementary material for full instructions). Participants were instructed to remain silent throughout the testing session and to do the experiment by themselves. The lecturer and a teaching assistant ensured that instructions were followed.

After receiving this information, participants provided demographic information (age, gender, degree program, nationality) and answered the 15 BPPQ for the first time (Fig 1). Afterwards, the lecturer introduced the students to the performer (Fig 1). After witnessing the demonstration, participants were asked to complete the two final parts of the online questionnaires, i.e. the event interpretation questions and the BPPQ questions for the second time (Fig 1). Finally, the magician debriefed participants about the true purpose of the study and explained the real method behind the effect (Fig 1.)

## Results

### Does the pseudo-psychological demonstration change peoples’ beliefs in psychological principles?

We looked at whether the demonstration changed people’s beliefs in the psychological principles that the magician claimed to be using. Here, the responses on the BPPQ were used. Before performing the respective comparisons, we tested how consistently participants answered across the five principles by calculating Cronbach’s alpha. Before the magic demonstration, the internal consistency (Cronbach’s alpha) revealed acceptable to good internal consistency for PBQ Belief (0.696), PBQ Used (0.870), and PBQ General (0.859). After the magic demonstration, the internal consistencies were slightly higher (0.759 for PBQ Belief; 0.888 for PBQ Used; 0.904 for PBQ General).

**Fig 2:**
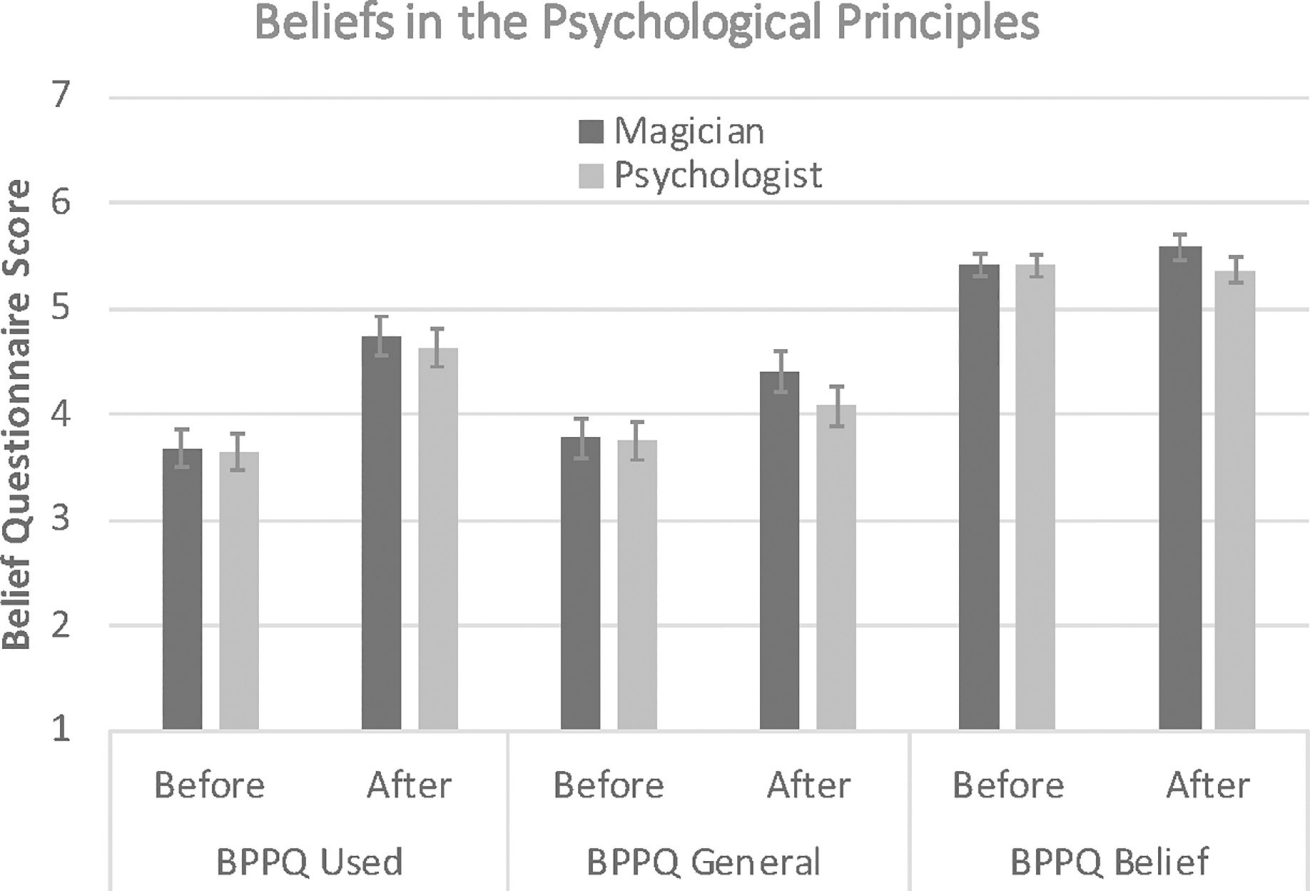
Mean PBBQ scores as a function of framing (magician vs. psychologist), type of PBBQ score before and after the performance. Error bars denote 95% Confidence Intervals.

Fig 2 shows the mean ratings for mean BPPQ Belief, BPPQ Used, and BPPQ General scores as a function of framing (magician, psychic), and time (before, after the demonstration). The respective ANOVA with BPPQ type (Belief, Used, General) and time as within-group factor and framing as between group factor on BPPQ scores found a significant main effect of PBBQ type, F(2, 176) = 178, p < .001, η^2^ = .67, time, F(1, 176) = 22.9, p < .001, η^2^ = .21, but not framing, F(1, 88) = 1.90, p = .46, η^2^ = .006. The ANOVA showed a significant time by BPPQ type interaction, F(2, 176) = 24.2, p < .001, η^2^ = .22. Post-hoc t-tests comparing the respective BPPQ type scores before and after the performance showed that whilst the demonstration did not change participants’ beliefs in the principle itself (BPPQ Belief), t(89) = .64, p = .54, Cohen’s d = 0.084, it significantly increased their beliefs that these principles were used in the performance (BPPQ Used), t(89) = 6.72, p < .001, Cohen’s d = 0.860, and that these principles can be used more generally (BPPQ General), t(89) = 3.24, p < .001, Cohen’s d = 0.379.

There were no significant PBBQ type by framing, F(2, 176) = 0.19, p = .83, η^2^ = .002, or time by framing interactions, F(2, 176) = 0.80, p = .38, η^2^ = .009, or a PBBQ type by framing by time interaction, F(2, 176) = 0.34, p = .71, η^2^ = .004.

### Correlations between BPPQ scores and event interpretation scores

The next analysis tested whether people’s BPPQ scores would correlate with their event interpretation scores (see also [14, 15]. Very few participants considered religious or psychic interpretations. Thus, we focused on trickery and psychological interpretations (see Table 1). Spearman correlation coefficients showed that BPPQ type scores correlated amongst themselves (Table 1). Neither the trickery nor the psychological interpretation scores correlated with any of the BPPQ type scores before the demonstration (Table 1). After the demonstration, higher psychological explanation scores correlated with higher BPPQ Belief, BPPQ General and BPPQ Used scores. After the demonstration, the trickery explanation scores did not correlate with any of the BPPQ type scores.

**Table 1:** Correlation table.

		Prior to the demonstration					After the demonstration		
		PB Belief	PB General	PB Used	Trickery	Psychology	PB Belief	PB General	PB Used
Prior to the demonstration	PB Belief	1	.303**	.210*	-0.051	0.132	0.19	0.114	0.184
	PB General		1	.625**	0.126	0.006	.232*	.406**	.352**
	PB Used			1	-0.016	-0.088	0.171	.255*	.282**
	Trickery				1	-0.035	-0.059	0.001	-0.027
	Psychology					1	.406**	.389**	.489**
After the demonstration	PB Belief						1	.607**	.596**
	PB General							1	.763**
	PB Used								1

Spearman correlation coefficients and significant results (indicated by asterisks

* = < .05

** p < .0005)

for BPPQ type scores before and after the demonstration and event interpretation scores (trickery, psychological)

### Does framing influence how people interpret a pseudo-psychological demonstration?

**Fig 3:**
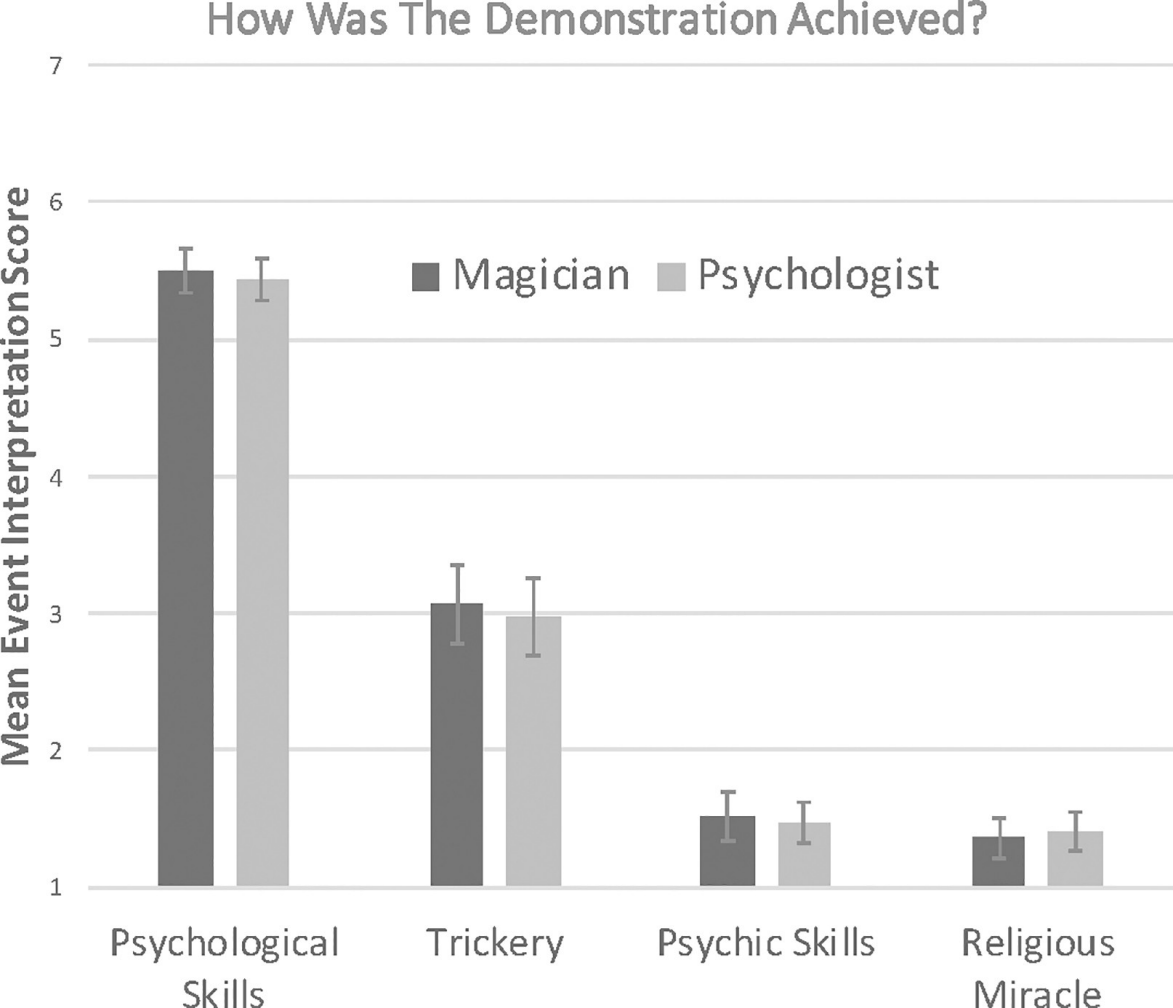
Mean event interpretation scores as a function of framing (magician vs. psychologist) for each of the skills. Error bars denote 95% Confidence Intervals.

This final analysis looked at whether framing influenced participants’ interpretation of the demonstration. Fig 3 shows the mean Likert ratings for how they thought the demonstration had been achieved, as a function of framing.

An ANOVA with skill (psychic, trickery, psychological, religious miracle) as a within-subject factor and framing (magician, psychologist) as a between-subject factor on event interpretation scores, found a significant main effect of skill, F(1, 88) = 59.1, p < .001, η^2^ = .40, but no significant main effect of framing, F(1, 88) = 0.049, p = .82, η^2^ = .001, or skill by framing interaction, F(1, 88) = 0.31, p = .58, η^2^ = .004. The framing did not influence how participants interpreted the demonstration. The psychological interpretation was the most popular (Fig 3). Bonferroni corrected t-tests revealed that the psychological interpretation scores were significantly higher than any of the other interpretation scores (all ps < 0.001). In addition, the trickery interpretation scores were significantly higher than the psychic and religious interpretation scores (all ps < .001). All remaining paired comparisons were not significant (all p-values > .05).

## Discussion

This paper tested the impact of a pseudo-psychological demonstration on people’s beliefs in implausible psychological principles. Our findings are clear and somewhat unnerving. Witnessing pseudo-psychological demonstrations significantly increased people’s beliefs that it was possible to 1) read a person’s mind by observing micro expressions, psychological profiles or muscle-reading, and 2) effectively prime a person’s decisions through subtle suggestions. Before the demonstration, the average observer was relatively uncertain as to whether these skills can be effectively used to determine which hand a person is holding a coin in. After seeing the demonstration, there was a significant increase in their beliefs, and the average observer now agreed more strongly that it was possible. Witnessing the demonstration also increased their beliefs in whether these principles can be used more generally to read a person’s thoughts in different situations.

The current findings contrast previous results using a similar paradigm, but testing psychic beliefs when seeing such impossible events of paranormal nature [14, 15]. Whilst the pre-existing beliefs determined the extent to which participants considered the demonstration to be a true psychic demonstration, the demonstration did not change their beliefs in the paranormal. In the current study, however, beliefs in such pseudo-psychological explanations were embraced, and we can conclude that people’s beliefs in scientifically plausible phenomena are more malleable. Moreover, whilst there were no significant correlations between the pre-demonstration beliefs and the extent to which participants attributed the demonstration to psychological skills, event interpretation significantly correlated with their beliefs in the pseudo-psychological principles after witnessing the demonstration. These correlations further support the view that witnessing the pseudo-psychological demonstration changed people’s beliefs in these principles.

Magicians have played an important role in debunking fraudulent psychics who use magic tricks to fake spiritual phenomena [8]. As a common rule, the magician does not intend to mislead the public. They often use disclaimers to inform the audience that what they are watching is a magic trick. However, our results show that knowledge about the nature of the performer–whether he was a magician or psychologist—did not influence people’s beliefs about how the demonstration was achieved, and neither did it influence their beliefs in the principles themselves.

One could argue that our framing manipulation was too subtle to have a substantial impact on participants’ reasoning about the event. Participants were told on three occasions that the performer was either a member of a magic or psychology society, but the University context may have provided legitimacy to the demonstration. However, in some of our previous research we asked participants to write down the person’s profession (psychic or magician) prior to watching the demonstration, and the impact was still negligible [19]. It was only once we explicitly told people how the trick was done, that the framing started to have a significant impact [20].

Magic is a popular form of entertainment, and pseudo-psychological demonstrations are frequently performed and witnessed by millions of people. These demonstrations may inadvertently mislead people’s beliefs about psychology. For example, magicians often use tricks and deception to enhance the perceived effectiveness of hypnotic suggestions. Stage hypnosis has played an important role in shaping beliefs about hypnosis. For example, after watching a hypnosis show on stage, a large proportion of participants claimed that the hypnotists had full control over his subjects [21], but this belief is not supported by empirical evidence [22].

Our results demonstrate that pseudo-psychological demonstrations can have a significant impact on perpetuating false beliefs in scientific principles, but they also raise some important questions about the wider impact of misinformation. Misinformation has a remarkable impact on people’s memories for past events [23] and their understanding of the events themselves [24]. Moreover, with the rise of the ‘fake news’ phenomena, recent research has investigated how people respond to false information that was later corrected [25]. In such studies, participants are presented with information that is initially presumed to be true, but is later corrected [26]. Results showed that corrections are rarely fully effective [26]. That is, despite being corrected, people rely on information they know to be false, a phenomenon known as continued-influence effect [27]. Thus, even when people know that what they are seeing may not be real, it can have a profound impact on their beliefs (see also [28]).

The influence of fictional narratives on our beliefs provide striking evidence, of how people often fail to consider the wider context in which the misinformation is presented. Intuitively, we should discount factual claims that are encountered in a fictional narrative. However, narrative theorists [29, 30] suggest that narratives are by default accepted as truthful and since the decoupling of fact from fiction requires cognitive effort, fictional information can potentially influence our beliefs. Indeed, a recent meta-analysis [31] concluded that there was no significant difference between the persuasive effect of factual and fictional narratives, which shows that misinformation in the context of fiction can influence attitudes and beliefs.

In our post-truth era, the boundaries between truth and misinformation have been eroded, and it is often difficult to distinguish between fact and fiction [10, 11]. One of the key features of this post-truth misinformation is that “fake news” is often very convincing. Clever editing and special effects are used to doctor online videos. Fake viral videos can spread across social network platforms, often viewed by millions of people. Rather than simply reading about a fake event, people are now witnessing the illusory event with their own eyes. Most past research on misinformation has presented participants with factually incorrect texts or verbal information. Very little is known about the impact of these more convincing forms of misinformation. As we have demonstrated here, stage magic provides a powerful tool to study the impact that highly realistic, yet entirely fake evidence, has on our beliefs and attitudes.

## Supporting information

S1 Text Questionnaire English. Belief in Psychological Principles Questions translated.
(DOCX)

S2 Text Framing instructions. Framing instructions used for the two groups.
(DOCX)

S3 Text Text. **Questionnaire original**. Belief in Psychological Principles Questions original.
(DOCX)

## References

[1] Kuhn G. Experiencing the impossible: The science of magic Cambridge, MA: MIT Press; 2019.

[2] Parris BA, Kuhn G, Mizon GA, Benattayallah A, Hodgson TL. Imaging the impossible: An fMRI study of impossible causal relationships in magic tricks. Neuroimage. 2009;45(3):1033–9. 10.1016/j.neuroimage.2008.12.036 WOS:000264378400038. 19166943 10.1016/j.neuroimage.2008.12.036 PMC2680974

[3] Leddington J. The experience of magic. The journal of aesthetics and art criticism. 2016;74(3):253–64.

[4] Rensink RA, Kuhn G. A framework for using magic to study the mind. Frontiers in Psychology. 2015;5 10.3389/fpsyg.2014.01508 25698983 10.3389/fpsyg.2014.01508 PMC4313584

[5] Lamont P, Steinmeyer J. The Secret History of Magic: The True Story of the Deceptive Art New York: Penguin Random House; 2018.

[6] Christopher M. The illustrated history of magic New York: Carroll & Graf Publishers; 2006.

[7] Wiseman R, Greening E, Smith M. Belief in the paranormal and suggestion in the seance room. Brit J Psychol. 2003;94(3):285–97. 10.1348/000712603767876235 . 14511544 10.1348/000712603767876235

[8] Marks DF. The psychology of the psychic Amherst, New York: Prometheus Books; 2000.

[9] Lamont P. Extraordinary beliefs: A historical approach to a psychological problem Cambridge: University Press; 2013.

[10] Legare CH, Evans EM, Rosengren KS, Harris PL. The coexistence of natural and supernatural explanations across cultures and development. Child development. 2012;83(3):779–93. 10.1111/j.1467-8624.2012.01743.x 22417318 10.1111/j.1467-8624.2012.01743.x

[11] Legare CH, Gelman SA. Bewitchment, biology, or both: The co‐existence of natural and supernatural explanatory frameworks across development. Cognitive Sci. 2008;32(4):607–42. 10.1080/03640210802066766 21635349

[12] Thomas C, Didierjean A. Magicians fix your mind: How unlikely solutions block obvious ones. Cognition. 2016;154:169–73. 10.1016/j.cognition.2016.06.002. 27318598

[13] Benassi VA, Singer B, Reynolds CB. Occult Belief—Seeing Is Believing. Journal for the Scientific Study of Religion. 1980;19(4):337–49. ISI:A1980KY59500002.

[14] Mohr C, Koutrakis N, Kuhn G. Priming psychic and conjuring abilities of a magic demonstration influences event interpretation and random number generation biases. Frontiers in Psychology. 2015;5 10.3389/fpsyg.2014.01542 25653626 10.3389/fpsyg.2014.01542 PMC4300903

[15] Lesaffre L, Kuhn G, Abu-Akel A, Rochat D, Mohr C. Magic Performances–When Explained in Psychic Terms by University Students. Frontiers in Psychology. 2018;9(2129). 10.3389/fpsyg.2018.02129 10.3389/fpsyg.2018.02129 PMC6232384 30459687

[16] Subbotsky E. Magical thinking in judgments of causation: Can anomalous phenomena affect ontological causal beliefs in children and adults? Brit J Dev Psychol. 2004;22:123–52. ISI:000220480900007.

[17] Woolley JD. Thinking about fantasy: Are children fundamentally different thinkers and believers from adults? Child Development. 1997;68(6):991–1011. 10.2307/1132282 WOS:A1997YL12000001. 9418217

[18] Subbotsky E. Children's and adults' reactions to magical and ordinary suggestion: are suggestibility and magical thinking psychologically close relatives? Br J Psychol. 2007;98(Pt 4):547–74. Epub 2007/10/13. 10.1348/000712606X166069 . 17931466 10.1348/000712606X166069

[19] Mohr C, Lesaffre L, Kuhn G. Exposure to magic. An experimental approach to test adult belief formation? Science of Magic Association Conference; London2017.

[20] Wicht C, Mohr C, Simmons K, Thomas C, Lesaffre L, G, editors. Explain paranormal events—they are influenced by pre-existing beliefs and availability of alternative explanations. Asscociation for the Scientific Study of Conscisousness; 2018; Krakau.

[21] Crawford HJ, Kitner-Triolo M, Clarke SW, Olesko B. Transient positive and negative experiences accompanying stage hypnosis. Journal of abnormal psychology. 1992;101(4):663 1430605 10.1037//0021-843x.101.4.663

[22] Nash MR, Barnier AJ. The Oxford handbook of hypnosis: Theory, research, and practice: Oxford University Press; 2012.

[23] Loftus EF, Pickrell JE. The formation of false memories. Psychiatric annals. 1995;25(12):720–5.

[24] Loftus EF, Palmer JC. Reconstruction of automobile destruction: An example of the interaction between language and memory. Journal of verbal learning and verbal behavior. 1974;13(5):585–9.

[25] Lewandowsky S, Ecker UKH, Cook J. Beyond Misinformation: Understanding and Coping with the “Post-Truth” Era. Journal of Applied Research in Memory and Cognition. 2017;6(4):353–69. 10.1016/j.jarmac.2017.07.008.

[26] Chan M-pS, Jones CR, Jamieson KH, Albarracín D. Debunking: A Meta-Analysis of the Psychological Efficacy of Messages Countering Misinformation. Psychological Science. 2017;28(11):1531–46. 10.1177/0956797617714579 . 28895452 10.1177/0956797617714579 PMC5673564

[27] Lewandowsky S, Ecker UKH, Seifert CM, Schwarz N, Cook J. Misinformation and Its Correction:Continued Influence and Successful Debiasing. Psychological Science in the Public Interest. 2012;13(3):106–31. 10.1177/1529100612451018 . 26173286 10.1177/1529100612451018

[28] Thomas C, Didierjean A, Kuhn G. It is magic! How impossible solutions prevent the discovery of obvious ones? The Quarterly Journal of Experimental Psychology. 2017.

[29] Busselle R, Bilandzic H. Fictionality and perceived realism in experiencing stories: A model of narrative comprehension and engagement. Communication Theory. 2008;18(2):255–80.

[30] Gilbert DT. How mental systems believe. American psychologist. 1991;46(2):107.

[31] Braddock K, Dillard JP. Meta-analytic evidence for the persuasive effect of narratives on beliefs, attitudes, intentions, and behaviors. Communication Monographs. 2016;83(4):446–67. 10.1080/03637751.2015.1128555

