# Supplementary material for: Fake science: The impact of pseudo-psychological demonstrations on people’s beliefs in psychological principles
Source: PLoS One. 2018 Nov 27;13(11):e0207629. doi: 10.1371/journal.pone.0207629 (PMC6258475; doi:10.1371/journal.pone.0207629)
Supplement: S1 Text — Belief in Psychological Principles Questions translated. (DOCX) [file pone.0207629.s001.docx]

**Belief in Psychological Principles Questions (BPPQ)**

These questions have been translated from Mandarin into English

Please indicate how much you agree or disagree with each of the statements below. To indicate your answer, use the numbers indicated below. There is no right or wrong answer. We assess your beliefs and attitudes. Thank you.

Strongly Disagree (1), Moderately Disagree (2), Slightly Disagree (3), Uncertain (4), Slightly Agree (5), Moderately Agree (6), Strongly Agree (7)

*Questions 1 – 5 were used to score BPPQ Belief, 6 – 10 BPPQ General and 11 – 15 BPPQ Used*

1. Micro expressions refer to very brief facial expressions that are involuntary and expose a person's real emotions. Micro expressions really exist.

2. Ideomotor actions refer to thought-induced, small, and involuntary physical reactions. Ideomotor actions really exist.

3. Personality refers to a person’s unique and consistent way of thinking and behaving. Personality really exists.

4. Suggestions refers to how one can influence a person to accept an idea, belief, or impulse in an uncritical way. Suggestions really exists.

5. Mind reading refers to the ability of one person to understand another person’s psychological state without normal means of communication. Mind reading really exists.

6. One can accurately decide whether a person is lying focusing on micro expression.

7. One can accurately interpret a person's thoughts focusing on ideomotor actions.

8. One can accurately predict a person's behavior focusing on personality.

9. One can effectively control a person's behavior focusing on suggestion.

10. One can fully understand a person's thoughts focusing on mind reading.

Now, imagine a scenario where a psychologist gives the participate a coin. Behind the back, the participant puts the coin in one hand. The psychologist has to find out the hand that holds the coin.

11. A well-trained psychologist can use micro expressions to accurately determine whether a person is lying. For example, psychologists can determine which hand holds a coin focusing on micro expressions.

12. A well-trained psychologist can use ideomotor actions to accurately interpret a person's thoughts. For example, psychologists can determine which hand holds a coin focusing on ideomotor actions).

13. A well-trained psychologist can use personality to accurately predict a person's behavior. For example, psychologists can determine which hand holds a coin focusing on personality.

14. A well-trained psychologist can use suggestion to affect a person's behavior. For example, psychologists can influence which hand holds the coin focusing on suggestion.

15. A well-trained psychologist can use mind reading to fully understand a person's thoughts. For example, psychologists can determine which hand holds a coin focusing on mind reading).
