# Supplementary material for: Fake science: The impact of pseudo-psychological demonstrations on people’s beliefs in psychological principles
Source: PLoS One. 2018 Nov 27;13(11):e0207629. doi: 10.1371/journal.pone.0207629 (PMC6258475; doi:10.1371/journal.pone.0207629)
Supplement: S2 Text — Framing instructions used for the two groups. (DOCX) [file pone.0207629.s002.docx]

**Supplementary material**

1. **Framing instructions**

Please note that the texts below were presented in Mandarin. Here, we present the English translations. The first translation was performed by the first author. After subsequent editing by the co-authors, the first author again verified the current final version.

Contextual framing instruction for the magic demonstration:

Title to the text: *“Tsinghua University* ***Magic*** *Association Questions”.* Participants read the following introduction: *“This experiment was initiated by the Tsinghua University* ***Magic*** *Association. The questions come in two parts, please answer them independently and seriously. During the experiment, please do not communicate with other students. After completing the first part, stop answering the questions until the member of the* ***Magic*** *Association finishes his demonstration. Then, continue to answer the second part. Thank you!”*

Participants read the following statement after they had finished the first part of the questions: *“Please stop answering the questions until the member of the* ***Magic*** *Association has finished his demonstration. Then, continue to answer the second part!”*

Contextual framing instruction for the psychology demonstration:

Title to the text: *“Tsinghua University* ***Psychology*** *Association Questions”. Participants* read the following introduction: *“This experiment was initiated by the Tsinghua University* ***Psychology*** *Association. The questions come in two parts, please answer them independently and seriously. During the experiment please do not communicate with other students. After completing the first part, stop answering the questions until the member of the* ***Psychology*** *Association finishes his demonstration. Then continue to answer the second part. Thank you!”*

Participants read the following statement after they had finished the first part of the questions: *“Please stop answering the questions until the member of* ***Psychology*** *Association has finished his demonstration. Then continue to answer the second part!”*

1. **Pseudo-Psychological Demonstration**

The performer started by taking a coin from his pocket. Then, he asked the lecturer to put the coin in her hand. The performer said “*Your dominant hand is the right hand, right*?”. She answered:” *Yes*.” Then he put the coin in her right hand and said:” *Now you can put it in either of your hands behind your back. Then hold out your fists in front of me. I can always tell you in which hand you hold the coin. Please do not play any trick like putting the coin into your pocket. Let’s try this four times*.”

Suggestion manipulation: Behind her back, the lecturer put the coin in one hand and then stretched out her arms with the fists in front of the performer. The performer used a secret device that informed him about the location of the coin. The performer approached her and said:” *Do you remember I mentioned right hand before*?” She said ”*Yes*”. He proceeded to say ”*This was a suggestion. By doing so, you would feel that the right hand would be too obvious to hide the coin in. Thus, I think you put the coin in your left hand*.” She opened her hands and he was right.

Psychology manipulation: The performer said ”*Let’s try it again*.” Behind her back, the lecturer put the coin again in one hand and stretched out her arms with the fists in front of the performer. The performer proceeded saying ”*This time, you will tell me whether you are a challenger or responder. Challengers will put the coin in the same hand as before, because they want to make a comeback. Responders, on the other hand, will put the coin into the other hand, because this choice seems less risky. Well, I think you are responder, so the coin should be in the right hand this time.”* She opened her hands and he was right.

Micro-expressions manipulation: The performer proceeded saying ”*Let’s try it again. You still have two more goes*”. Behind her back, the lecturer put the coin again in one hand and stretched out her arms with the fists in front of the performer. The performer approached her and said “*I will ask you in which hand you have put the coin. You have to say “no” for each hand. This way, you have to lie for one hand. I will try to catch your lie.*” Then he asked twice, once for each hand, whether the coin was in the respective hand. After her response, the performer said ”*I think, I know now in which hand you hold the coin. It is in your right hand, right?”* She opened her hands and he was right.

Muscle reading manipulation: The performer continued saying ”*This is your last time. Take some time to make your final decision*.” Behind her back, the lecturer put the coin again in one hand and stretched out her arms with the fists in front of the performer. The performer approached her and said ”*I will ask you to take a posture involving your hands. This will help me knowing in which hand you hold the coin. Cross your arms and put one hand on top of the other*.” After she had taken this posture he proceeded saying ”*People usually put the hand holding the coin below the other hand. They do not want to be too obvious. So… Wait! I think you will do the opposite. Open your upper hand please*.” She opened her hand and again he was correct.
