# Supplementary material for: Fake science: The impact of pseudo-psychological demonstrations on people’s beliefs in psychological principles
Source: PLoS One. 2018 Nov 27;13(11):e0207629. doi: 10.1371/journal.pone.0207629 (PMC6258475; doi:10.1371/journal.pone.0207629)
Supplement: S3 Text — Questionnaire original. Belief in Psychological Principles Questions original. (DOCX) [file pone.0207629.s003.docx]

**Belief in Psychological Principles Questions**

请在每个陈述的边上选择一个数字，来代表您多大程度上同意或者不同意那个陈述。这仅代表你自己的观点。

| 1=完全不同意 2=大致上不同意 3=有点不同意 4=不清楚 5=有点同意 6=大致上同意 7=完全同意  **Belief in principle (PBBQ Belief)** |
| --- |
| 1. 微表情（指非常短暂的面部表情，具有不自主性并会暴露一个人的真实情绪）真实存在 |
| 2. 观念运动（指由思想引发的、轻微的、不自觉的身体反应）真实存在 |
| 3. 人格（指人所具有的与他人相区别的独特而稳定的思维方式和行为风格）真实存在 |
| 4. 暗示（指影响一个人不加批判地接受一个想法、信念或冲动）真实存在 |
| 5. 读心术（指在与他人交流过程中，当即能够了解对方的心理状态和想法）真实存在  **Principles used in the demonstration (PBBQ General)** |
| 6. 通过微表情可以准确判断一个人是否说谎 |
| 7. 通过观念运动可以准确解读一个人的想法 |
| 8. 通过人格可以准确预测一个人的行为 |
| 9. 通过暗示可以完全影响一个人的行为 |
| 10. 通过读心术可以完全了解一个人的想法 |
| **现在请想象这样一个场景：一个心理学家交给被试一枚硬币，这名被试可以在身后将硬币放在任何一只手里，而心理学家要做的是猜出硬币在哪只手里。(PBBQ General)** |
| 11. 训练有素的心理学家可以通过微表情准确判断一个人是否在说谎（例如心理学家可以通过微表情猜出硬币在哪只手里） |
| 12. 训练有素的心理学家可以通过观念运动准确读出一个人的想法（例如心理学家可以通过观念运动猜出硬币在哪只手里） |
| 13. 训练有素的心理学家可以通过人格准确预测一个人看似随机的行为（例如心理学家可以通过人格预测被试会把硬币放在哪只手里） |
| 14. 训练有素的心理学家可以通过暗示完全影响一个人看似随机的决定（例如心理学家可以通过暗示影响被试将硬币放在哪只手里） |
| 15. 训练有素的心理学家可以通过读心术完全了解一个人的想法（例如心理学家可以通过读心术知道被试将硬币放在哪只手里） |

第二部分

9. 对于刚才的实验所实现的方法，请在每个陈述的边上选择一个数字，来代表您多大程度上同意或者不同意那个陈述。这仅代表你自己的观点。

1=完全不同意 2=大致上不同意 3=有点不同意 4=不清楚 5=有点同意 6=大致上同意 7=完全同意

| (1) 通过通灵的或超自然的力量实现的 |  |
| --- | --- |
| (2) 通过一般的魔术实现的 |  |
| (3) 通过心理学技巧实现的 |  |
| (4) 通过宗教奇迹实现的 |  |

10. 请在每个陈述的边上选择一个数字，来代表您多大程度上同意或者不同意那个陈述。这仅代表你自己的观点。

| 1=完全不同意 2=大致上不同意 3=有点不同意 4=不清楚 5=有点同意 6=大致上同意 7=完全同意 |
| --- |
| 1. 微表情（指非常短暂的面部表情，具有不自主性并会暴露一个人的真实情绪）真实存在 |
| 2. 观念运动（指由思想引发的、轻微的、不自觉的身体反应）真实存在 |
| 3. 人格（指人所具有的与他人相区别的独特而稳定的思维方式和行为风格）真实存在 |
| 4. 暗示（指影响一个人不加批判地接受一个想法、信念或冲动）真实存在 |
| 5. 读心术（指在与他人交流过程中，当即能够了解对方的心理状态和想法）真实存在 |
| 6. 通过微表情可以准确判断一个人是否说谎 |
| 7. 通过观念运动可以准确解读一个人的想法 |
| 8. 通过人格可以准确预测一个人的行为 |
| 9. 通过暗示可以完全影响一个人的行为 |
| 10. 通过读心术可以完全了解一个人的想法 |

| 11. **现在请想象这样一个场景：一个心理学家交给被试一枚硬币，这名被试可以在身后将硬币放在任何一只手里，而心理学家要做的是猜出硬币在哪只手里。**  **1=完全不同意 2=大致上不同意 3=有点不同意 4=不清楚 5=有点同意 6=大致上同意 7=完全同意** |
| --- |
| 1. 训练有素的心理学家可以通过微表情准确判断一个人是否在说谎（例如心理学家可以通过微表情猜出硬币在哪只手里） |
| 2. 训练有素的心理学家可以通过观念运动准确读出一个人的想法（例如心理学家可以通过观念运动猜出硬币在哪只手里） |
| 3. 训练有素的心理学家可以通过人格准确预测一个人看似随机的行为（例如心理学家可以通过人格预测被试会把硬币放在哪只手里） |
| 4. 训练有素的心理学家可以通过暗示完全影响一个人看似随机的决定（例如心理学家可以通过暗示影响被试将硬币放在哪只手里） |
| 5. 训练有素的心理学家可以通过读心术完全了解一个人的想法（例如心理学家可以通过读心术知道被试将硬币放在哪只手里） |

问卷结束！感谢您的参与！
